# Supplementary material for: Innate and adaptive immune traits are differentially affected by genetic and environmental factors
Source: Nat Commun. 2017 Jan 5;8:13850. doi: 10.1038/ncomms13850 (PMC5227062; doi:10.1038/ncomms13850)
Supplement: Supplementary Information — Supplementary Figures 1-3 [file ncomms13850-s1.pdf]

**Panel 1: T Cell Differentiation**	**Panel 2: T Cell Activation**	**Panel 3: T Cell Polarization**	**Panel 4: NK Cell Differentiation**	**Panel 5: Stem cells; Innaptive Differentiation**	**Panel 6: B Cell Differentiation**	**Panel 7: Myeloid Cells**
Viability	Viability	Viability	Viability	Viability	Viability	Viability
CD3	CD3	CD3	CD2	CD34	CD19	CD14
CD4	CD4	CD4	CD3		CD20	CD11c
CD8	CD8	CD8	CD4	CD3		CD123
			CD16	CD4	IgA	HLA-DR
CD27	CD25	CD45RA	CD56	CD8	IgD	
CD45RA	CD127	CCR7			IgG	CD1c
CCR7	CD39	PD1	CCR7	CD1d	IgM	CD141
	CD73		CD62L			
CD28		CXCR3	CD158a	TCR-Vγ9	CD5	CD8
CD31	CD45RA	CXCR5	CD158b	TCR-Vδ1	CD10	CD16
CD57	CD38	CCR4	CD314	TCR-Vδ2	CD21	CD32
CD95	CCR5	CCR6	CD335		CD24	CD64
CD127	PD1	CCR10	CD337	CD27	CD27	CD83
CD244	HLA-DR	CD161		CD28	CD95	CD274
				CD45RA	CD38	HLA-DR
				CCR7		
				CCR5		

Supplementary Figure 1. Immunophenotyping panels used in this study. Seven 13 or 14-color panels were optimized to enumerate and phenotype different leukocyte populations from cryopreserved PBMC. All seven panels were applied to all subjects using staining temperatures optimized for each panel. Reagents are roughly ordered by: major lineage-defining markers; sub-lineage defining markers; and then markers to assess differentiation stages, activation state, and/or functional capacity.

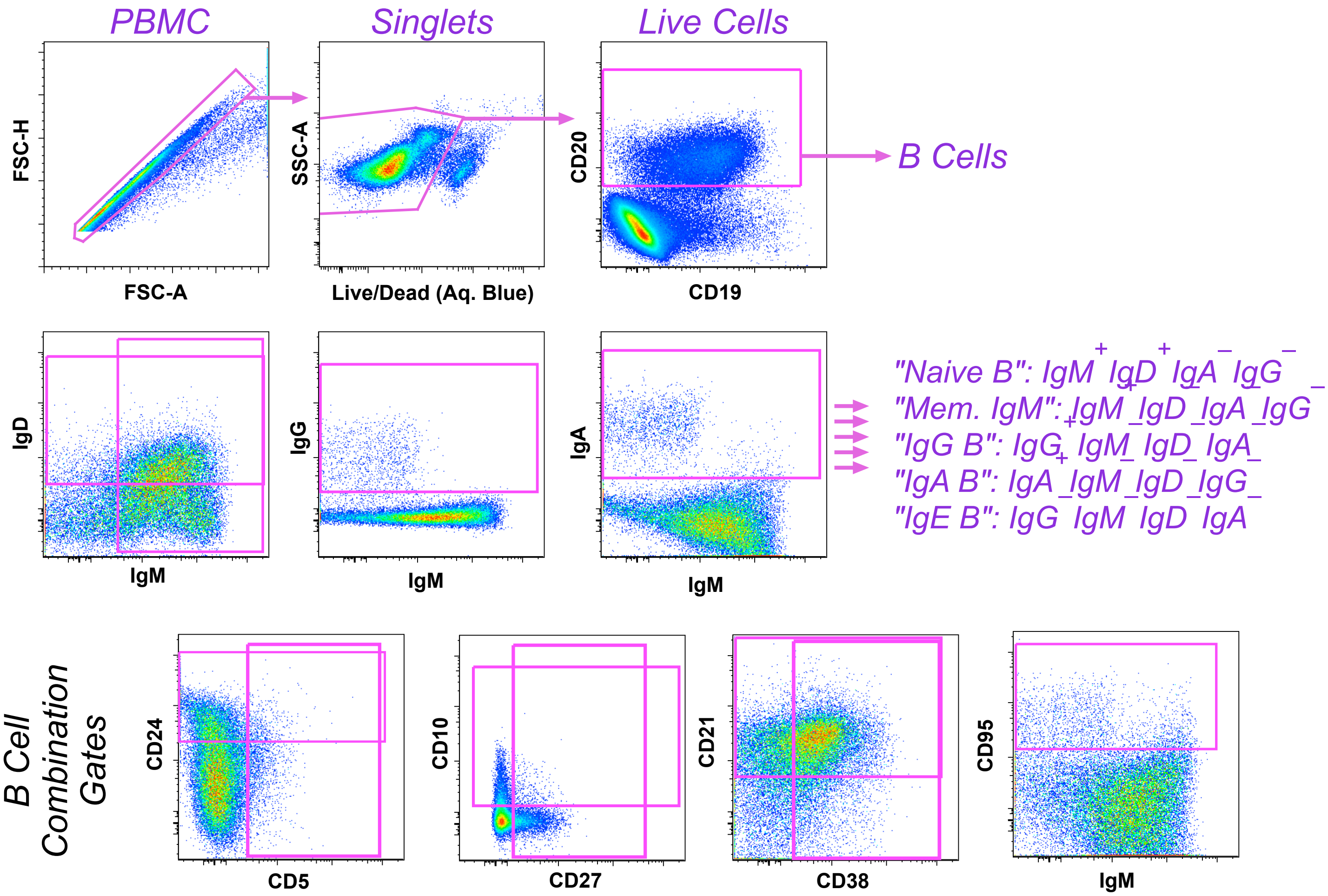

Supplementary Figure 2. Gating for B cell subsets. A different gating strategy was used for Panel 6 than originally described[8]. B cells were broadly identified as CD20+ live lymphocytes (top). Within these cells, gates identifying isotype expression were combined to as-purely-as-possible identify naïve and memory B cells; within the latter, IgM, IgG, IgA, and putative IgE-expressing cells were gated. Within each of these five, all combinations of the seven markers (positive, negative, or ignored) were created by Boolean gating as previously described[8]. This results in 2,187 CSF traits for each of the five isotype-defined subsets.

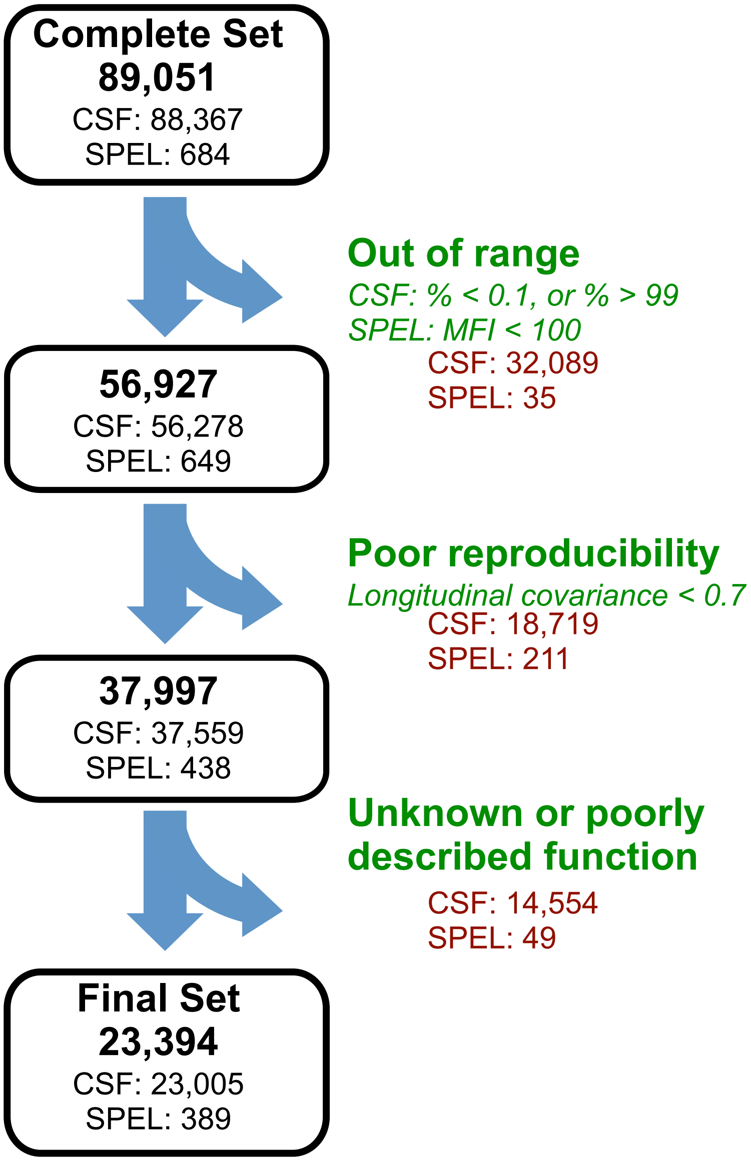

Supplementary Figure 3. Trait QC workflow. Structured equation modelling was performed on a robust subset of all traits. Three different filters (shown in green) were applied in succession to the original 89,051 traits to result in the final 23,394. The last filter was a subjective, eliminating cell populations such as $CD4^{+}CD8^{+}$ (double positive) or $CD4^{-}CD8^{-}$ (double negative) T cells from consideration.
